# Immediate Analgesic Effect of Acupuncture in Patients With Primary Dysmenorrhea: A fMRI Study

**DOI:** 10.3389/fnins.2021.647667

**Published:** 2021-05-24

**Authors:** Yanan Wang, Jing Xu, Qing Zhang, Qi Zhang, Ya Yang, Wei Wei, Xiaoli Guo, Fanrong Liang, Siyi Yu, Jie Yang

**Affiliations:** ^1^Acupuncture and Tuina School, Chengdu University of Traditional Chinese Medicine, Chengdu, China; ^2^People’s Hospital of Yuxi City, Yuxi, China; ^3^Chongqing Traditional Chinese Medicine Hospital, Chongqing, China

**Keywords:** primary dysmenorrhea, immediate analgesic effect, fMRI, acupuncture, pain

## Abstract

Primary dysmenorrhea (PDM) is a common gynecological disease characterized by lower abdominal pain. Acupuncture is considered a good alternative therapy for PDM. However, the central mechanism of the analgesic effect of acupuncture is largely unknown. In this study, eligible patients were randomized into the real and sham acupuncture groups using a computer-generated, permuted block randomization method. The study cohort comprised 34 patients: 19 in the real acupuncture group and 15 in the sham acupuncture group. The clinical characteristics of the patients during their menstrual period were collected, and imaging scans were performed during the first 3 days of the patients’ menstrual period. We analyzed task and resting functional magnetic resonance imaging (fMRI) data to investigate the potential central mechanism of the immediate effect of acupuncture intervention on the intensity of PDM pain. The task fMRI study found that the rostral anterior cingulate cortex (rACC) and right supplemental motor area were activated during real acupuncture. Using the resting-state functional connectivity (FC) method, we found a post- versus pre-treatment change in the FC of the rACC and left precentral gyrus in the comparison of real acupuncture versus sham acupuncture. In addition, the FC of the rACC–left precentral gyrus at baseline was negatively correlated with short-term analgesia, while the change in the FC of the rACC–left precentral gyrus was positively correlated with short-term analgesia after acupuncture treatment. These findings support the importance of rACC–left precentral gyrus resting-state FC in the modulation of the intensity of PDM pain through acupuncture, which may shed light on the central mechanism of acupuncture in the treatment of PDM.

## Introduction

Primary dysmenorrhea (PDM) is a common gynecological disease characterized by pain in the lower abdomen (Ruoff and Lema, 2003). The onset of PDM often occurs at the age of 20 years or younger after the ovulatory cycle becomes established (Lentz et al., 2012). PDM reportedly affects 45–95% of menstruating women, and severe episodes affect 10–25% of reproductive-aged women (Iacovides et al., 2015). PDM has become the main cause of recurrent absenteeism in young women during their menstrual period (Pitangui et al., 2013). Non-steroidal anti-inflammatory drugs and oral contraceptives are widely used in the treatment of PDM (Dawood, 2006). However, these standard drugs have a poor therapeutic effect and are associated with adverse effects (Harada et al., 2011; Marjoribanks et al., 2015; Oladosu et al., 2018). Therefore, there is an urgent need to identify a safe and effective method with which to manage the symptoms of PDM.

Recently, acupuncture has been considered a good alternative therapy for PDM in patients who fail to respond to oral analgesic drugs (Sriprasert et al., 2015). A series of clinical studies have confirmed the beneficial effect of acupuncture in improving pain due to PDM (Ma et al., 2013; Armour et al., 2017; Shetty et al., 2018). In addition, a meta-analysis reported that acupoint stimulation has more significant effects on PDM than non-acupoint stimulation (Chung et al., 2012). Moreover, studies on the analgesic effect of single acupoint stimulation have proved that acupuncture at Sanyinjiao (SP6) effectively relieves dysmenorrhea (Liu et al., 2014; Hu and Liu, 2019), especially resulting in an immediate effect in PDM (Liu et al., 2011; Shi et al., 2011; Mohammadi et al., 2019). However, the underlying mechanism by which acupuncture exerts an immediate analgesic effect in patients with PDM remains unclear.

In recent years, neuroimaging techniques have been extensively used to investigate the mechanism by which acupuncture achieves analgesia. A large number of functional magnetic resonance imaging (fMRI) studies have demonstrated that the analgesic effect of acupuncture may be related to the responses of pain-related brain regions (Li et al., 2014; Zhao et al., 2014; Yan et al., 2020). Moreover, the analgesic mechanism of acupuncture in PDM is related to the regulation of endogenous opioids in the pain modulation system and pain-related brain network (Ren et al., 2012). Thus, the probable mechanism of the immediate analgesic effect of acupuncture in PDM is via modulation of the brain regions related to pain.

The acupuncture process is complicated, and task-state fMRI studies have found that twirling acupuncture manipulation at real and sham acupoints results in different activation effects on the cerebral cortex in healthy people (Fang J. et al., 2004; Fang J.L. et al., 2004). However, it is unclear whether this difference affects the outcome of acupuncture in patients with PDM. To investigate this, we used a block design to divide patients into groups that underwent acupuncture at real or sham acupoints × twirling acupuncture and retention acupuncture. Task-state fMRI was utilized to verify whether the immediate analgesic effect and brain response to twirling acupuncture differed between acupuncture modalities (needling at acupoints or non-acupoints). Furthermore, we used resting-state fMRI to examine the effects of acupuncture on the functional connectivity (FC) of different brain areas and to identify whether the changes in brain FC could be used to evaluate changes in clinical pain intensity. We hypothesized that the combined task- and resting-state brain function data would result in different central response characteristics due to acupoint and non-acupoint stimulation under different needle manipulation techniques, thus explaining part of the central mechanism of the immediate analgesic effect of acupuncture in PDM.

## Materials and Methods

### Patients

Forty-four patients with PDM were recruited. The inclusion criteria were as follows: (1) right-handed nulliparous women aged 18–30 years; (2) meeting the diagnostic criteria for PDM of the Society of Obstetricians and Gynecologists of Canada (Burnett and Lemyre, 2017); (3) normal menstrual cycle (27 to 32 days); (4) fulfillment of a menstrual cycle dysmenorrhea diary; (5) average pain intensity score of ≥4 on a visual analog scale (VAS) for three consecutive menstrual cycles; (6) fMRI scan performed on days 1–3 of menstruation and a VAS pain score of ≥ 4 before the scan; and (7) responsive to acupuncture and able to attain the feeling of *De qi*. The exclusion criteria were as follows: (1) dysmenorrhea caused by organic gynecological disease; (2) history of cardiovascular and/or cerebrovascular diseases and/or neuropsychiatric disorders; (3) intake of painkillers or sedatives within 1 week before the fMRI scan; (4) contraindications to fMRI. All patients provided written informed consent for study participation. The study was approved by the Sichuan Traditional Chinese Medicine Regional Ethics Review Committee before trial commencement. Nine patients dropped out during the follow-up period. A final total of 34 patients (19 in the real acupuncture group and 15 in the sham acupuncture group) completed all the clinical assessments and imaging scans and received 8 weeks of real or sham acupuncture treatment.

Patients were instructed to take no analgesics other than ibuprofen during the study period. Participants were permitted to take ibuprofen sustained-release capsules (*Tianjin Smith Kline & French laboratories, Ltd., Tianjin, China; SFDA approval number: H20013062*) as rescue medicine for unbearable menstrual cramps; the details, including dosage, medication time, and remission time were reported to the researchers and recorded in the case report form.

#### Randomization and Blinding

Eligible patients were randomized into the real and sham acupuncture groups using a computer-generated, permuted block randomization method. Because of the particularities of acupuncture, it is difficult to achieve blinding. To minimize the influences of subjective factors from participants and researchers on the research results, we blinded the patients, assessors, and statisticians to the groupings.

#### Data Acquisition

##### Demographic and clinical data

The patients’ baseline demographic data (age, height, weight, and disease duration) were collected. VAS scores were recorded before and after the fMRI scan to evaluate the degree of dysmenorrhea immediately before and after acupuncture.

##### fMRI scan procedure

The study was performed in the MRI room of the Affiliated Hospital of Chengdu University of TCM. The fMRI scan was acquired using a 3.0-T GE Signa MR750 system (GE Healthcare, Milwaukee, WI, United States). All patients were scanned within the first 3 days of their menstrual period. Patients were instructed not to drink strong tea, coffee, or alcohol for 24 h before the MRI scan. After a 20-min break, the patients entered the MRI room in a calm state. Patients were asked to keep their eyes closed and remain awake but avoid psychological activity as much as possible during the scanning process. A head restraint was used to limit head movement, and earplugs were provided to reduce the noise.

The three scanning steps in turn were T1-weighted gradient-echo imaging, rest 1, and rest 2. High-resolution T1-weighted gradient-echo images were obtained for anatomical reference using the following parameters: repetition time (TR) = 2530 mm, echo time (TE) = 3.39 ms, field of view (FOV) = 256 mm × 256 mm, flip angle = 7°, slice thickness = 1 mm, resolution = 256 × 256. The resting-state functional images were obtained using an echo-planar imaging sequence with the following parameters: TR = 2000 ms, TE = 30 ms, FOV = 240 mm × 240 mm, flip angle = 90°, slice thickness = 4 mm, resolution = 64 × 64.

##### Acupuncture procedure

**FIGURE 1:**
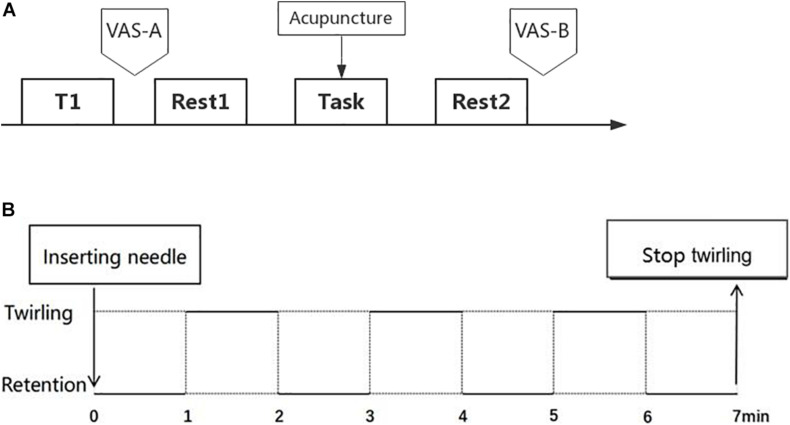
Schematic diagram of the fMRI experimental process. **(A)** Structural-image scan (T1), function-image scan (rest1, rest2, and task), the paradigm of intervention (acupuncture), and evaluation of pain intensity (VAS-A and VAS-B). **(B)** In the task-state scanning, the acupuncture operation of the twirling and retention were performed alternately, with each condition for 1 min, and the total operating time lasted 7 min.

After the first two scans (T1-weighted gradient-echo imaging and rest 1), the scanning was suspended and patients were instructed to keep their whole body still, especially the head. Patients then received acupuncture at the corresponding acupoints or non-acupoints in accordance with their grouping. The following two types of conditions were applied: bilateral SP6 stimulation, and stimulation at a non-acupoint (without known clinical effects) located midway between SP6 and the Urinary Bladder Meridian. The patients had not previously received acupuncture and could not distinguish which acupoints were being stimulated. Needles were perpendicularly inserted to a depth of 1–1.5 cun. The acupuncturists twirled the needles bidirectionally from 90° to 180° to induce the *De qi* sensation. The duration of acupuncture was 7 min, during which the needles were retained and twisted alternately. The time at which the needles were inserted was designated as “0.” The needles were inserted at 1, 3, 5, and 7 min, twirled at 2, 4, and 6 min (at a rate of approximately 120 times per minute), and then left in place at the end of 7 min (Figure 1). After the completion of acupuncture, the rest 2 scan was performed with the needles retained. The whole process was continuous. The procedure was performed by licensed and experienced acupuncturists. The disposable stainless steel acupuncture needles did not cause any safety problems under the nuclear magnetic environment.

#### Statistical Analysis

All statistical analyses were performed using SPSS Statistics V21.0. Continuous variables with normal distribution were presented as the mean and standard deviation. The significance level used for statistical analysis with two-tailed testing was 5%. For normally distributed quantitative data, the *t* test was used to detect differences between the real and sham acupuncture groups; the Mann–Whitney test was used for non-normally distributed data. To explore the effect of acupuncture on analgesia, the two-sample *t* test was used to compare the pre- to post-treatment change in pain intensity (VAS score) between the real and sham acupuncture groups.

#### fMRI Data Preprocessing and Analysis

All fMRI data were preprocessed using the Matlab toolkit SPM8^1^. To maintain the stability of the data, the images from the first five time points were discarded during data preprocessing. Then, all the scanned images were aligned with the middle image and corrected. When the head movement was corrected, the images with a head movement angle of greater than 2 mm or 2° in any direction were deleted. For spatial registration, T1-weighted images of the patients were matched with their respective functional images, and the images were uniformly segmented and resampled. The retained data were processed with spatial normalization based on the Montreal Neurological Institute space. Finally, the images were smoothed spatially with a Gaussian kernel at a full width at half maximum of 6 mm.

First, the one-sample *t* test was used to obtain the brain activity of each patient under each condition (real-twirling, real-retention, sham-twirling, and sham-retention). Second, the paired *t* test was used to obtain the fMRI signal change differences between the twirling and retention conditions within the same group. Third, a 2 × 2 analysis of variance was performed to compare the twirling and retention fMRI signal changes between the real and sham acupuncture groups (full factorial analysis in SPM8 second-level analysis) to yield an interest mask of the associated brain regions (Supplementary Figure 1). A voxel-wise threshold of *p* < 0.005 with a false discovery rate-corrected cluster level of *p* < 0.05 was applied in the task fMRI analysis; a small volume correction was used to correct for multiple comparisons using 3dClustSim (voxel level, *p* < 0.005; cluster level, *p* < 0.05) if the affected brain regions belonged to the pain matrix.

#### Functional Connectivity Analysis

Based on the region of interest as the seed point, seed-based FC was assessed in the resting-state fMRI scans during the pre- and post-treatment phases. Pearson correlation analysis was used to calculate the correlation (*r* value) between the seed region and each region of the brain. The *r* value was then converted into a *Z* value close to the normal distribution via Fisher *Z* transformation to represent the strength of the connection between the two brain regions.

#### Correlation Analysis

To investigate the correlation between the intergroup difference in the FC change (post- versus pre-treatment) and the short-term analgesic effect of acupuncture, we explored the potential correlation between the FC in characteristic brain regions and the VAS score based on Pearson’s correlation coefficient. We also explored the correlation between the brain areas activated under task-state fMRI and short-term analgesia.

## Results

### Patient Characteristics

A total of 34 patients completed this trial, comprising 19 in the real acupuncture group and 15 in the sham acupuncture group. The demographic data of all patients are shown in Table 1. There were no significant differences between the two groups in age, height, weight, and disease duration (*p* > 0.05).

**TABLE 1:** Baseline patient characteristics and average pain intensity before and after acupuncture treatment.

	Group		*t*	*P* value*
	RA (*n* = 19)	SA (*n* = 15)		
Age (years)	25.37 ± 2.41	24.20 ± 2.01	1.51	0.141
Height (cm)	160.68 ± 4.10	159.33 ± 4.75	0.92	0.365
Weight (kg)	51.11 ± 4.36	50.50 ± 4.38	0.40	0.691
Illness duration (months)	98.68 ± 45.75	87.53 ± 29.26	0.82	0.419
VAS-A	6.20 ± 1.06	5.91 ± 1.22	0.69	0.493
VAS-B	3.65 ± 1.04	4.45 ± 1.29	–1.89	0.069
VAS(B-A)	−2.55 ± 1.32	−1.45 ± 1.13	–2.33	0.027
VAS(B-A)/A	−0.41 ± 0.17	−0.25 ± 0.17	–2.74	0.010

Data are given as mean ± standard deviation. *p < 0.05 was considered statistically significant. RA, real acupuncture; SA, sham acupuncture; VAS, visual analog scale; VSA-A, VAS score before treatment; VAS-B, VAS score after treatment; VAS(B-A), change in the VAS score (post- minus pre-treatment); VAS(B-A)/A, ratio of the change in the VAS score [(post- minus pre-treatment)/pre-treatment].

The pre- and post-treatment VAS scores of the real and sham acupuncture groups are shown in Table 1. There were no significant differences between the two groups regarding the pre-treatment VAS score (*p* = 0.493) and post-treatment VAS score (*p* = 0.069), but the reduction in the pain intensity after treatment was significantly greater in the real acupuncture group than the sham acupuncture group. Furthermore, the pain intensity change [(post-treatment VAS score minus pre-treatment VAS score)/pre-treatment VAS score] was significantly greater in the real acupuncture group than the sham acupuncture group (*p* = 0.010).

#### Brain Region Activity

**FIGURE 2:**
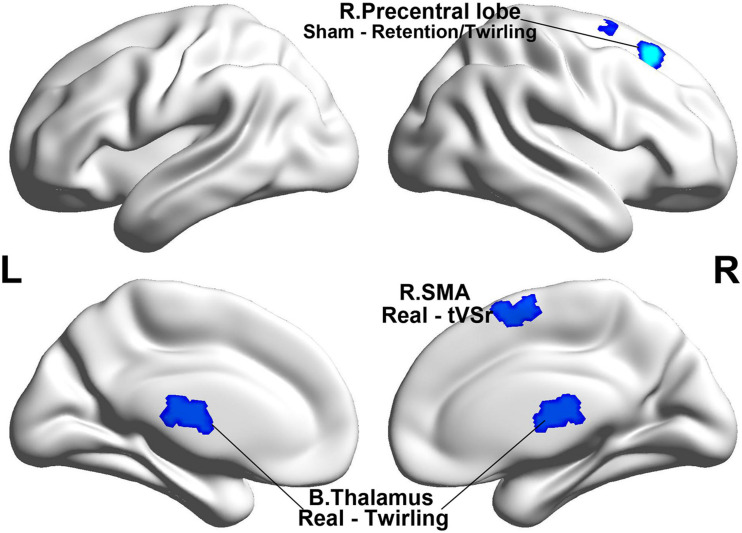
Brain negative activity in different conditions in each group. SMA, supplemental motor area; t VS r, twirling VS retention.

**FIGURE 3:**
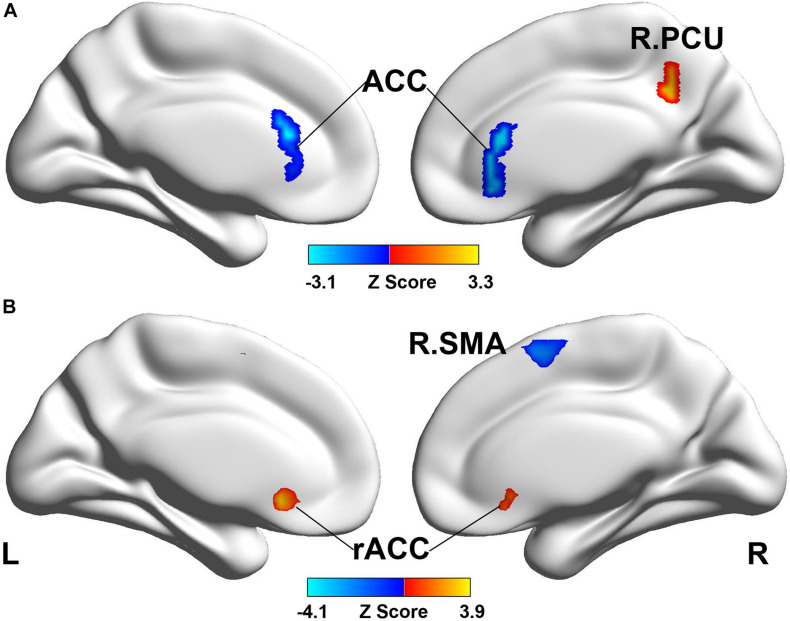
Brain activity in different conditions between real vs. sham treatment group. **(A)** The brain activity difference of retention condition and twirling condition between real vs. sham acupuncture treatment. **(B)** The brain activity difference of twirling vs. retention condition between real vs. sham acupuncture treatment. ACC, anterior cingulate cortex; PCU, precuneus; SMA, supplemental motor area; rACC, rostral anterior cingulate cortex.

Table 2 summarizes the task-state fMRI results that show the brain activity in each group and the intergroup differences in brain activity under each condition. Figure 2 shows the brain areas with no activity under different conditions in the real and sham acupuncture groups. The real acupuncture group showed no significant brain activity in the needle retention state, while bilateral thalamus activation was found in the needle twirling state; a comparison of the two conditions (retention versus twirling) revealed that the signal change occurred in the right supplemental motor area (SMA) of the brain. The sham acupuncture group showed brain activity in the right superior frontal gyrus (SFG) under both the retention and twirling states, with no difference in the activated brain area under the retention versus the twirling state. Under the retention condition, the brain activity significantly differed between the real and sham acupuncture groups in the anterior cingulate cortex (ACC), right precuneus (PCU), right SMA, and right middle frontal gyrus (MFG); under the twirling condition, the brain regions with significantly different activity in the real acupuncture group versus the sham acupuncture group were the right PCU and the ACC (Figure 3A). Notably, the ACC and PCU are the core nodes of the default mode network (DMN). The brain regions where the activity changes under the two conditions (retention versus twirling) differed between the real and sham acupuncture groups were the rostral ACC (rACC) and the right SMA (Figure 3B). In addition, there was a significant positive activity change in the rACC under the twirling versus the retention condition between the real and sham acupuncture groups.

**TABLE 2:** Task state-related brain activity in each group and intergroup activity differences under each condition.

Group condition	Area (BA)	Voxel size	MNI coordinates (RAI)			Peak *Z* Score
			*x*	*y*	*z*	
**RA retention**	**No significant activity**					
RA twirling	B. thalamus	131	0	−15	9	−3.51
RA twirling vs. retention	R.SMA(6)	148	18	3	63	−3.49
SA twirling	R.SFG(8)	123	15	15	45	−3.80
SA retention	R.SFG(8)	114	18	21	45	−3.87
SA twirling vs. retention	No significant activity					
Retention RA vs.SA	ACC(32/24)	66*	3	30	−6	−3.29
	R.PCU(7)	35*	9	−42	69	3.29
	R.SMA/SFG(6)	65*	18	21	45	3.33
	R.MFG/dlPFC(6)	36*	30	9	51	3.89
Twirling RA vs. SA	R.PCU(31)	32*	15	−51	36	3.27
	ACC(24)	38*	0	24	18	−3.03
Twirling/Retention vs. RA/SA	rACC(25)	62*	−3	21	−9	3.84
	R.SMA(6)	246	15	3	60	−4.04

*Calculated using a small volume correction for multiple comparisons using 3dClustSim (voxel level, *p* < 0.005; cluster level, *p* < 0.05). BA, Brodmann area; RA, real acupuncture; SA, sham acupuncture; SMA, supplemental motor area; SFG, superior frontal gyrus; ACC, anterior cingulate cortex; PCU, precuneus; MFG, middle frontal gyrus; dlPFC, dorsolateral prefrontal cortex; rACC, rostral anterior cingulate cortex.

#### Seed-Based Functional Connectivity Results and Their Correlation With the Pain Score

**FIGURE 4:**
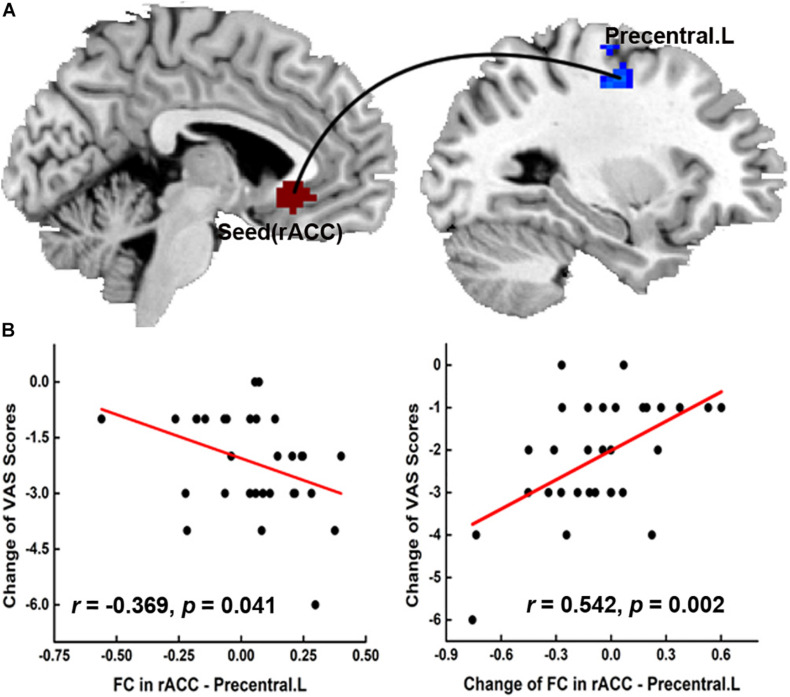
The seed-based functional connectivity results and clinical significance. **(A)** The significant group difference FC change (post- vs. pre-) between RA and SA group was found in FC between rACC and left precentral gyrus. **(B)** The FC of rACC-Precentral.L in the baseline was negatively correlated with the short-time pain analgesia, while the change of FC in rACC-Precentral.L was positively correlated with the short-time pain analgesia in PDM patients after acupuncture treatment. FC, functional connectivity; rACC, rostral anterior cingulate cortex; VAS, visual analog scale.

As shown in Figure 4, we chose the rACC as the seed point to use as the average time course of the reference regions for comparison with every other voxel in the brain. After calculating the resting-state FC, we found a significant pre- versus post-treatment difference in the FC between the rACC and the left precentral gyrus in the real acupuncture group versus the sham acupuncture group (Figure 4A). The resting-state scan showed that the FC of the rACC–left precentral gyrus at baseline was negatively correlated with the short-term analgesic effect based on the VAS pain score (*r* = −0.369, *p* = 0.041), while a positive correlation was observed between the change in FC of the rACC–left precentral gyrus and the short-term analgesic effect based on the VAS score (*r* = 0.542, *p* = 0.002) (Figure 4B).

## Discussion

In our study, the task-state fMRI results showed that twirling acupuncture (at both real and sham acupoints) caused different brain activities in pain-related brain areas, which indicates that there are multiple neural mechanisms involved in acupuncture. The DMN contributed greatly in the process of acupuncture. In addition, the pain intensity (scored by the VAS) was related to central features on resting-state fMRI. Our results suggest that the analgesic mechanism of acupuncture may be strongly linked to the FC of the rACC–left precentral gyrus.

### Task-State-Related Brain Activity in Each Group and Intergroup Differences in Brain Activity Under Each Condition

Our task-state fMRI results showed that the right SMA, right thalamus, and right SFG were activated under the twirling condition of acupuncture. The SMA plays a pivotal role in the translation of pain cognition (Puri et al., 2010); it expresses more significant regional cerebral blood flow under different pain modalities and is considered a potential biomarker of acute pain (Frölich et al., 2012). The brain response in the SMA is reportedly correlated with reduced pain following acupuncture stimulation (Maeda et al., 2013), suggesting that the SMA may mediate the signal transmission of acupuncture analgesia. Our study revealed deactivations in the SMA under the twirling acupuncture condition, which is in line with fMRI studies reporting that the SMA has decreased activity during acupuncture or transcranial stimulation (Tanaka et al., 2005; Deng et al., 2016).

Several studies have reported thalamus-related abnormal FC in PDM (Liu P. et al., 2017; Han et al., 2019). The thalamus, as part of the medial pain system, is significantly activated in response to the pain signal produced by painful stimuli (Derbyshire et al., 1997), which is mainly transmitted by the ascending pain pathway, and participates in the learning of the pain-related emotional response during the process of pain coding (McNally et al., 2011). The activation of the thalamus is positively correlated with the acupuncture-induced analgesic effect (Zhang et al., 2003; Gong et al., 2006). We speculate that this analgesic effect of acupuncture may be linked to the limbic–paralimbic–neocortical network modulation involving the thalamus (Fang et al., 2009). Moreover, the right SFG was activated by twirling acupuncture in the sham acupuncture group. This might be a result of the “placebogenic” effect of stimulation at non-acupuncture points (Howick, 2017), which occurs due to the unspecific effects of sham acupuncture (Madsen et al., 2009). Overall, the evidence suggests that the central analgesic effect of twirling acupuncture may be associated with mediation of the pain-related regions of the brain.

The present study found that the brain activity differed between the real and sham acupuncture groups in the ACC, right PCU, right SMA/SFG, and right MFG/dorsolateral prefrontal cortex. The ACC and PCU are the two core areas of the DMN (Fransson, 2005). Multiple fMRI studies have confirmed the presence of DMN-related abnormalities in PDM, involving structural abnormalities and aberrant intrinsic brain activities (Wu et al., 2016; Dun et al., 2017; Liu P. et al., 2017). Moreover, one tract-based analysis reported that the disruption of communication within the DMN and/or between the DMN and other pain-related brain networks induced by altered anatomical connections may be the central susceptibility leading to the progression of PDM into chronicity (Liu J. et al., 2017). A review focused on the central effects of acupuncture-induced analgesia indicated that the FC and activation pattern of the DMN are influenced by analgesic acupuncture (Otti and Noll-Hussong, 2012). Previous studies observed that patients with PDM have abnormal brain activity in the ACC, which might be associated with the PDM-related disturbance in pain modulation (Liu J. et al., 2017; Liu P. et al., 2017; Liu et al., 2018). Negative activation of the ACC inhibits the pain-related emotional response under the action of acupuncture (Hui et al., 2010), which proves that the ACC is an important brain region involved in acupuncture-induced analgesia. The PCU modulates chronic pain (Farmer et al., 2012) and acts on the autonomic nerve and descending inhibition systems to regulate the pain sensation caused by acute noxious stimulation (Kucyi et al., 2013). Abnormal changes in gray matter volume are observed in the PCU of patients with PDM (Tu et al., 2013; Liang et al., 2016; Chen et al., 2019). Moreover, patients with PDM have increased FC (Liu P. et al., 2017) and spontaneous brain activity (Jin et al., 2017) in the PCU, and acupuncture treatment significantly regulates this abnormal brain functional network connection (Cai et al., 2018). In addition, the SMA, SFG, right MFG, and dorsolateral prefrontal cortex were activated under task-state fMRI in the present study. The fMRI activation is more extensive in discriminative somatosensory and cognitive pain processing areas during verum acupuncture compared with sham acupuncture (Usichenko et al., 2015).

### Rostral Anterior Cingulate Cortex–Left Precentral Gyrus Functional Connectivity

A resting-state fMRI study confirmed the presence of abnormal ACC-related systematic FC in PDM-related disturbances (Liu et al., 2018). Furthermore, ACC-related resting-state FC is more prone to occur in women than men (Coulombe et al., 2016). In the present study, we found that real acupuncture significantly decreased the resting-state FC between the rACC and left precentral gyrus compared with sham acupuncture. The rACC mainly modulates internal pain responsiveness (Devinsky et al., 1995) and has been suggested to be involved in the FC within the mesocorticolimbic pathways encoding the effects of expectancy on pain (Tu et al., 2020). The precentral gyrus contributes to the provision of pain relief (Lefaucheur et al., 2001). An arterial spin labeling study reported aberrant precentral gyrus activity in patients with PDM (Zhang et al., 2019). Thus, a reduction in the rACC–left precentral gyrus resting-state FC may be the mechanism by which acupuncture affects pain modulation.

### Correlations Between the Rostral Anterior Cingulate Cortex–Left Precentral Gyrus Functional Connectivity and Pain Intensity

In this study, we combined task-state and resting-state fMRI data to identify the mechanism of the immediate effect of acupuncture intervention on the severity of pain due to PDM. We found a negative association between the predicted intervention effect and brain response in the real acupuncture group. Our findings indicate that the baseline rACC–left precentral gyrus FC may influence subsequent clinical responses involving the same network and provide a potential method with which to predict the response to acupuncture treatment. We also found that the change in FC of the rACC–left precentral gyrus was positively correlated with the short-term analgesic effect of acupuncture treatment in patients with PDM, which further endorsed the importance of rACC–left precentral gyrus resting-state FC in the modulation of the pain intensity of PDM through acupuncture. These findings suggest that the change in the rACC–left precentral gyrus resting-state FC may be the underlying central mechanism of the immediate analgesic effect of acupuncture.

### Study Limitations

This study has some limitations. The patients’ expectations of the outcome of acupuncture may have impacted the study results, although minimal communication was required during the study period. As the fMRI scans were performed within the first 3 days of the menstruation period, the compliance of some patients was affected and there were some dropouts during the follow-up period. Moreover, due to financial constraints, imaging data were not collected during the non-menstrual period. Finally, the sample size was small. The present findings require confirmation in further large-scale studies.

## Conclusion

The present study showed that twirling acupuncture (at both real and sham acupoints) resulted in different brain activities in pain-related brain areas, suggesting that acupuncture acts via multiple neural mechanisms. The task fMRI study found that the rACC and right SMA were activated during real acupuncture. The resting-state FC method showed that the post- versus pre-treatment change in FC between the rACC and the left precentral gyrus differed after real acupuncture versus sham acupuncture. In addition, the FC of the rACC–left precentral gyrus at baseline was negatively correlated with short-term analgesia, while the change in FC of the rACC–left precentral gyrus was positively correlated with the short-term analgesic effect of acupuncture treatment in patients with PDM. These findings support the importance of the rACC–left precentral gyrus resting-state FC in the modulation of the pain intensity of PDM through acupuncture, which may shed light on the central mechanism of acupuncture in the treatment of PDM.

## Data Availability Statement

The raw data supporting the conclusions of this article can be obtained on reasonable request from the corresponding author.

## Ethics Statement

This study was approved by Sichuan Traditional Chinese Medicine Regional Ethics Review Committee (approval number: 2013KL-033). All enrolled participants were clearly informed about the whole trial and provided written informed consent for study participation. This trial was performed in accordance with the principles of the Declaration of Helsinki.

## Author Contributions

JY, FL, and SY designed this trial and obtained the necessary funding. YW, QinZ, QiZ, YY, WW, and XG recruited patients and carried out this trial. SY analyzed the data and helped to revise the manuscript. YW and JX drafted and critically revised this manuscript. All authors discussed and approved the final manuscript for publication.

## Conflict of Interest

The authors declare that the research was conducted in the absence of any commercial or financial relationships that could be construed as a potential conflict of interest.

## Footnotes

1 http://www.fil.ion.ucl.ac.uk/spm/

## Supplementary Material

Supplementary Figure 1 Flow chart of fMRI study.
