# Supplementary figures and images for: Immediate Analgesic Effect of Acupuncture in Patients With Primary Dysmenorrhea: A fMRI Study
Source: Front Neurosci. 2021 May 24;15:647667. doi: 10.3389/fnins.2021.647667 (PMC8180846; doi:10.3389/fnins.2021.647667)

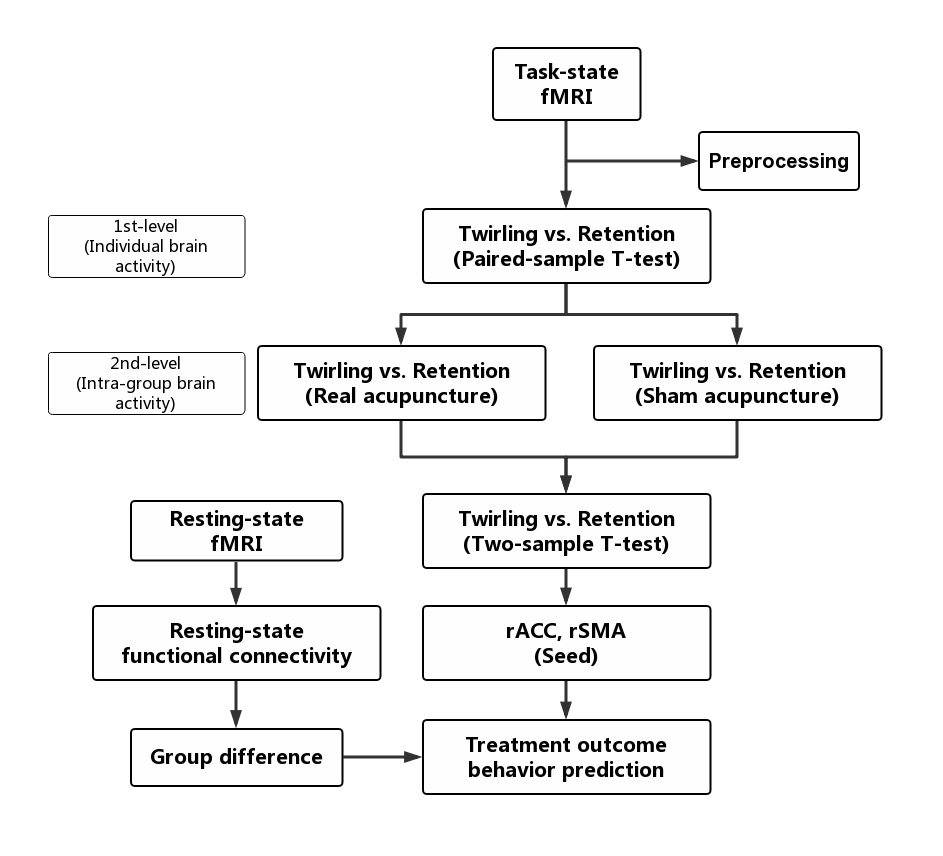

Supplement: Supplementary Figure 1 — Flow chart of fMRI study. [file Image_1.JPEG]
